# Supplementary figures and images for: Linker histone H1.8 inhibits chromatin binding of condensins and DNA topoisomerase II to tune chromosome length and individualization
Source: eLife. 2021 Aug 18;10:e68918. doi: 10.7554/eLife.68918 (PMC8416026; doi:10.7554/eLife.68918)

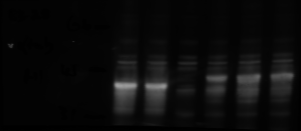

Supplement: Figure 1—source data 1. [file elife-68918-fig1-data1.zip › Fig 1/Figure 1B_H18.tif]

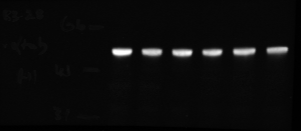

Supplement: Figure 1—source data 1. [file elife-68918-fig1-data1.zip › Fig 1/Figure 1B_tubulin.tif]

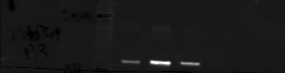

Supplement: Figure 1—source data 1. [file elife-68918-fig1-data1.zip › Fig 1/Figure 1E_CAPG_1.tif]

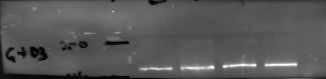

Supplement: Figure 1—source data 1. [file elife-68918-fig1-data1.zip › Fig 1/Figure 1E_CAPG_2.tif]

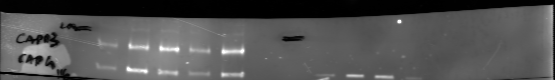

Supplement: Figure 1—source data 1. [file elife-68918-fig1-data1.zip › Fig 1/Figure 1E_CAPG_3.tif]

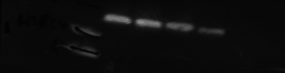

Supplement: Figure 1—source data 1. [file elife-68918-fig1-data1.zip › Fig 1/Figure 1E_H2B-CAPG_1.tif]

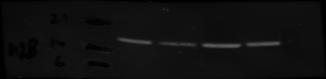

Supplement: Figure 1—source data 1. [file elife-68918-fig1-data1.zip › Fig 1/Figure 1E_H2B-CAPG_2.tif]

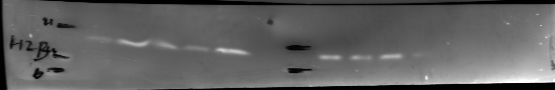

Supplement: Figure 1—source data 1. [file elife-68918-fig1-data1.zip › Fig 1/Figure 1E_H2B-CAPG_3.tif]

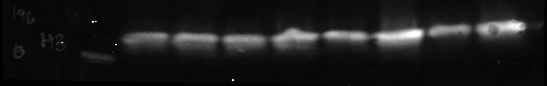

Supplement: Figure 1—source data 1. [file elife-68918-fig1-data1.zip › Fig 1/Figure 1E_H3-TOP2A_2.tif]

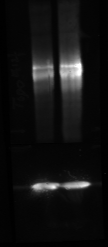

Supplement: Figure 1—source data 1. [file elife-68918-fig1-data1.zip › Fig 1/Figure 1E_TOP2A_1.tif]

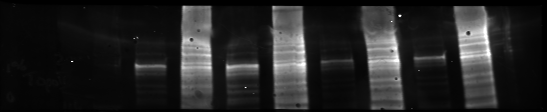

Supplement: Figure 1—source data 1. [file elife-68918-fig1-data1.zip › Fig 1/Figure 1E_TOP2A_2.tif]

**Figure 1B**

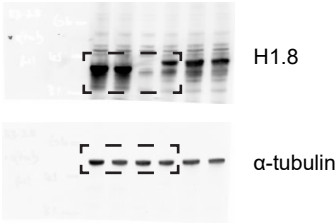

**Figure 1E (CAP-G)**

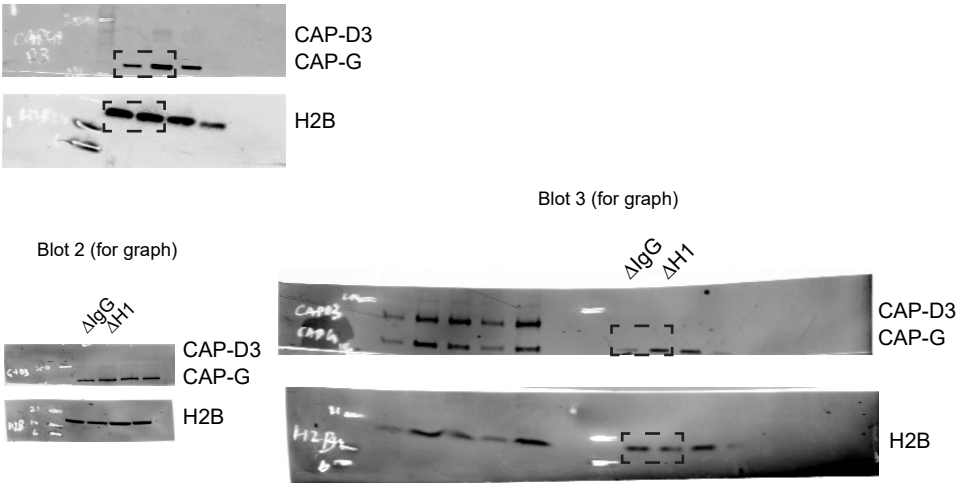

**Figure 1E (TOP2A)**

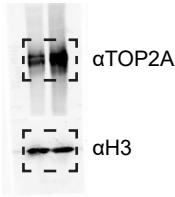

Blot 2 (for graph)

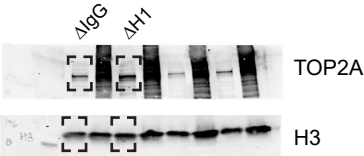

**Figure 1H**

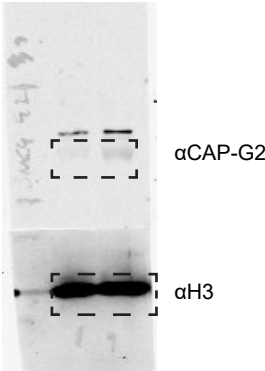

Supplement: Figure 1—source data 1. [file elife-68918-fig1-data1.zip › Fig 1/Figure 1-gels, blots.pdf]

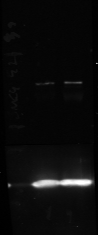

Supplement: Figure 1—source data 1. [file elife-68918-fig1-data1.zip › Fig 1/Figure 1H.tif]

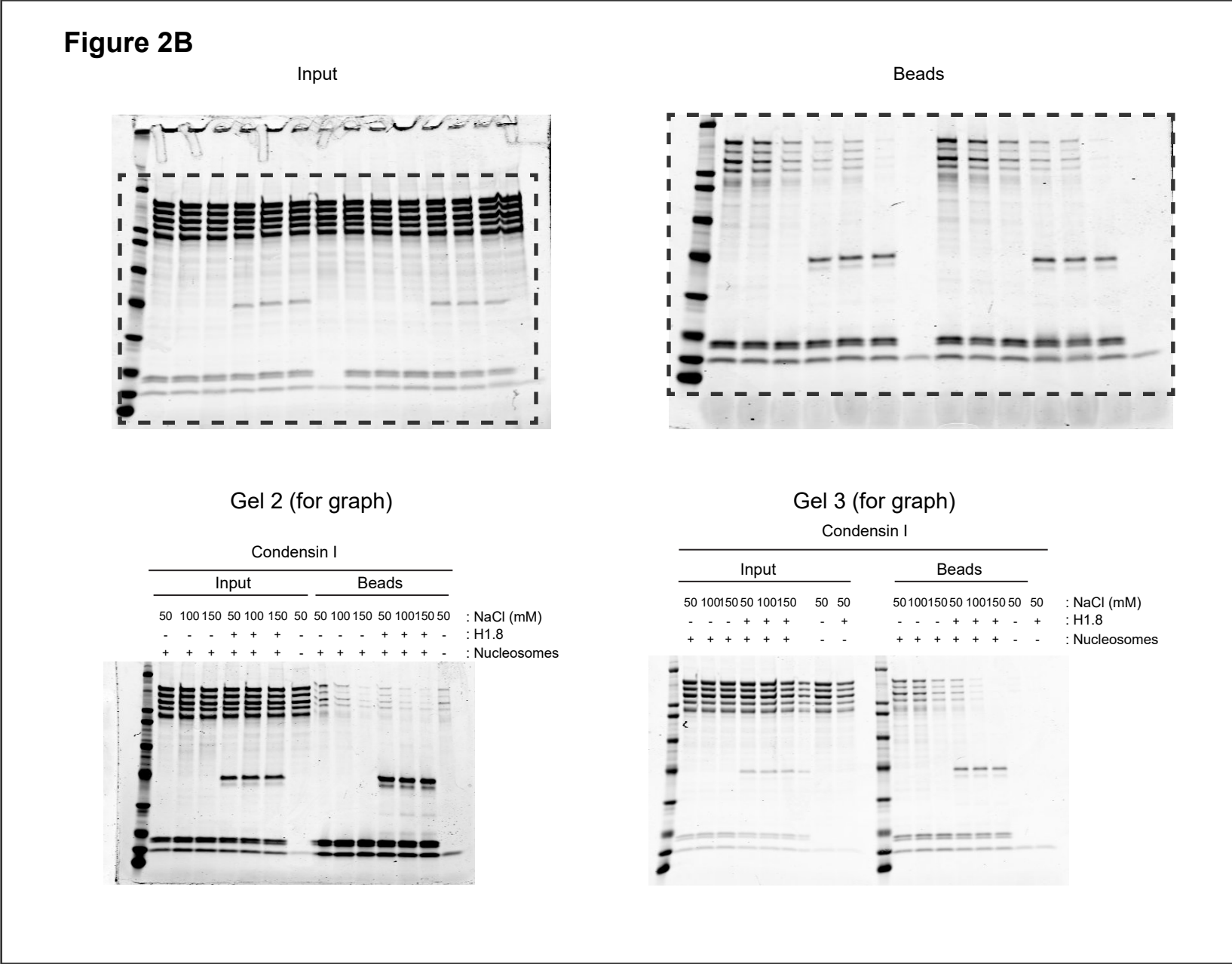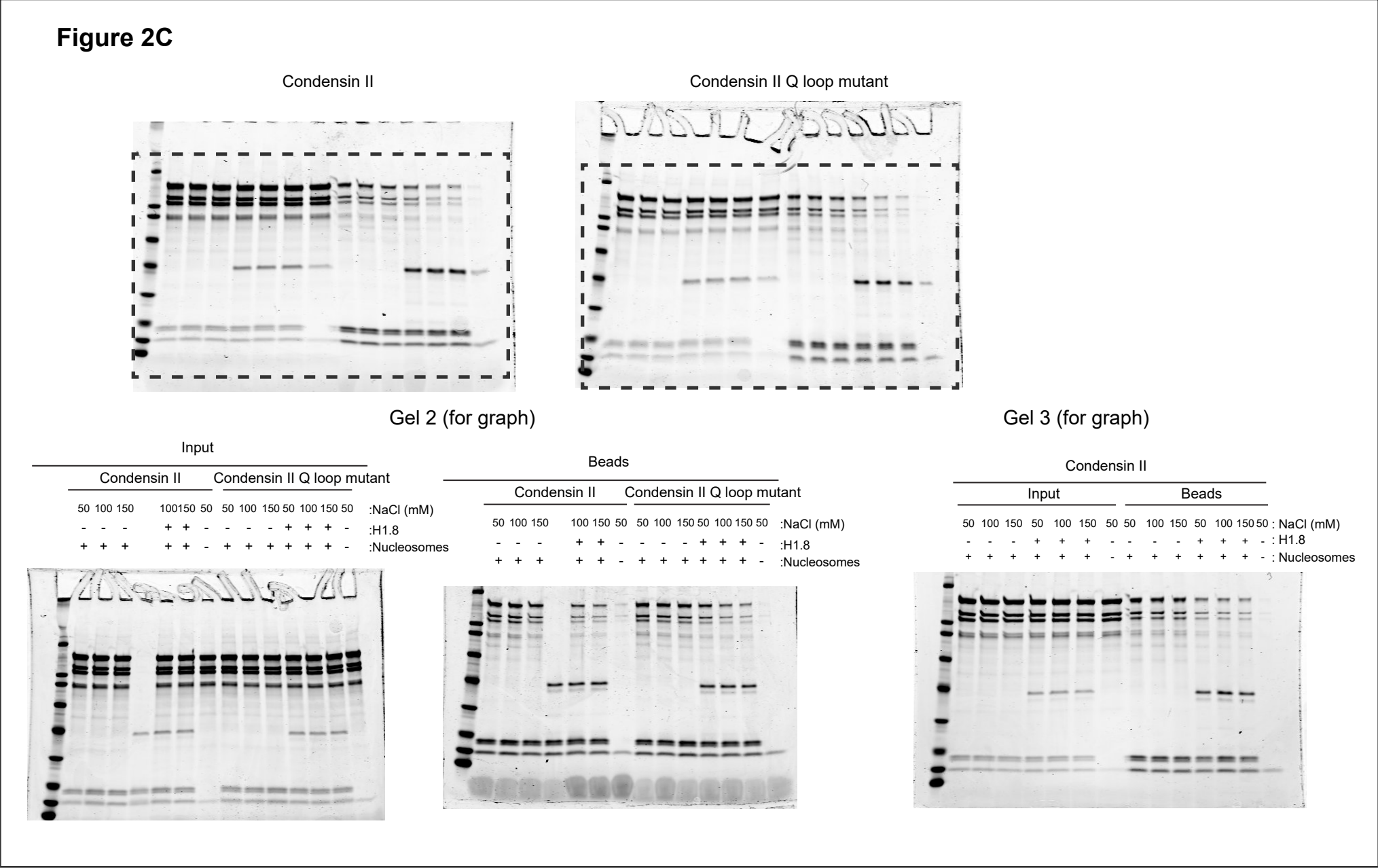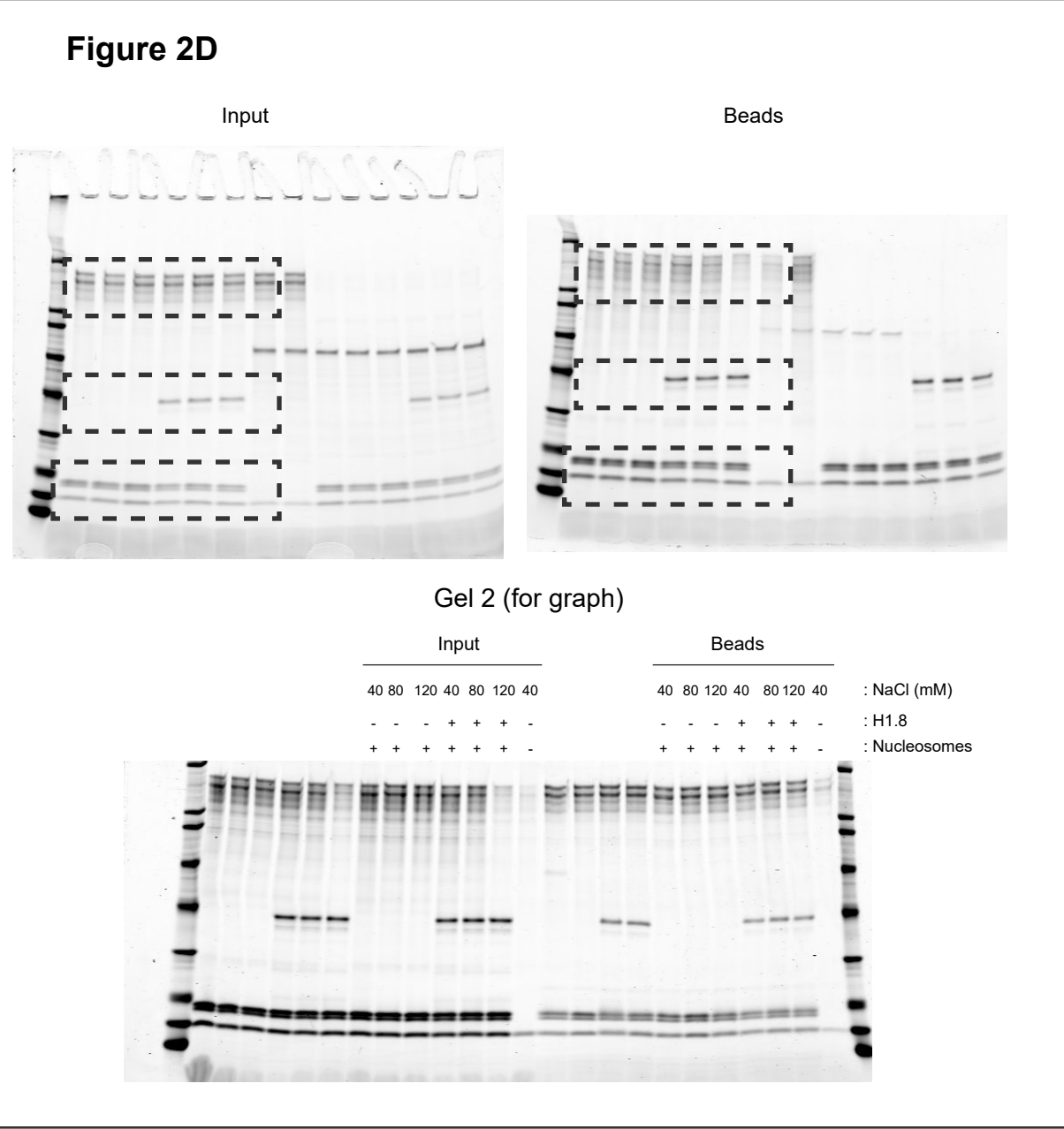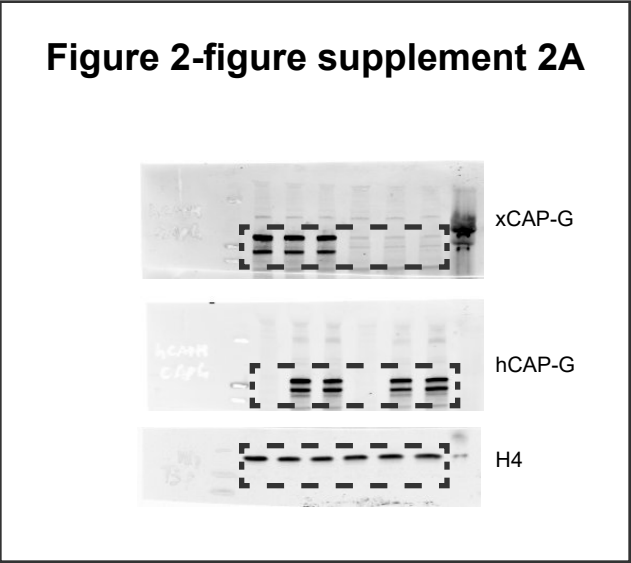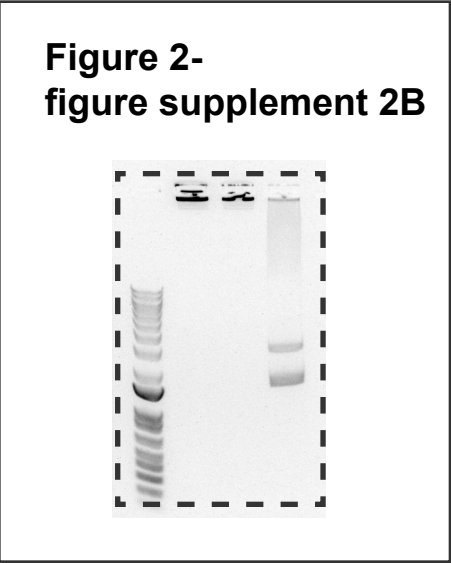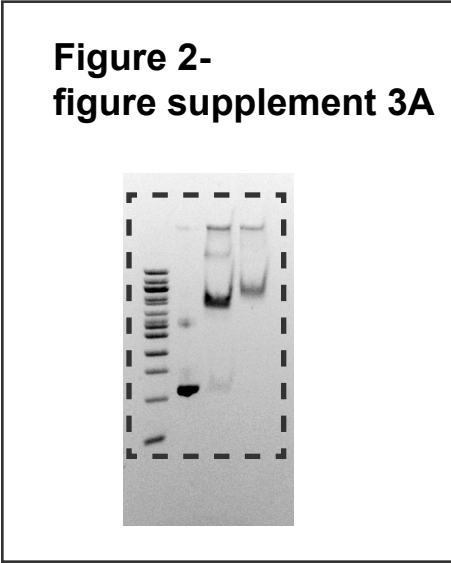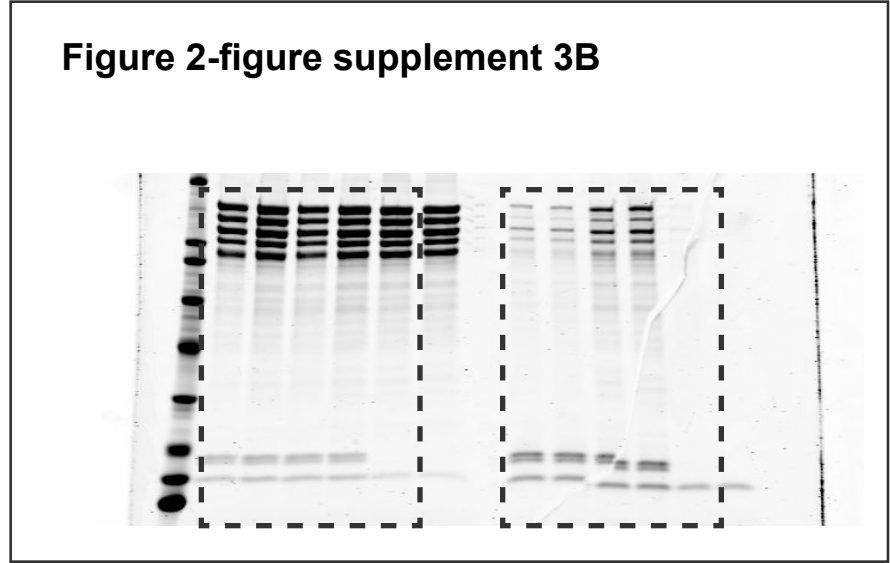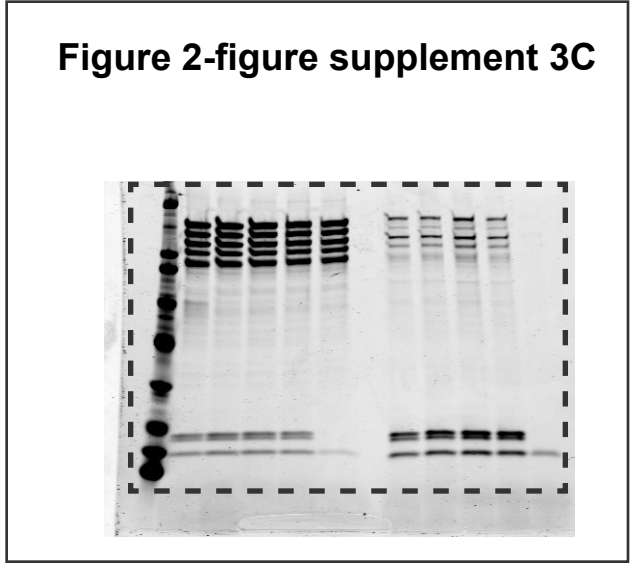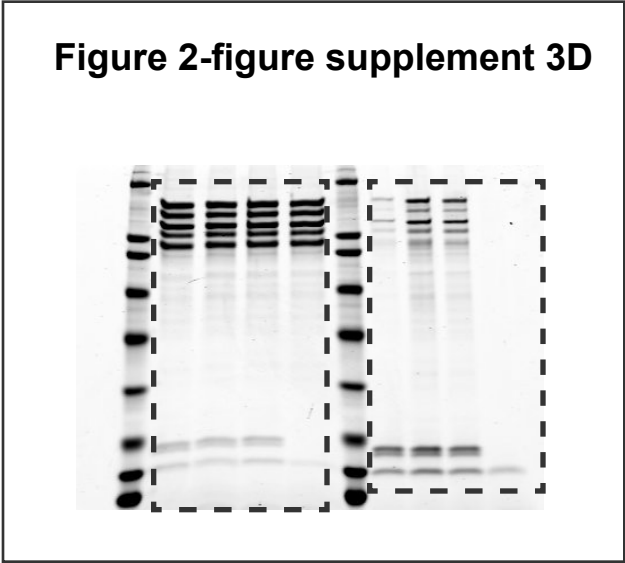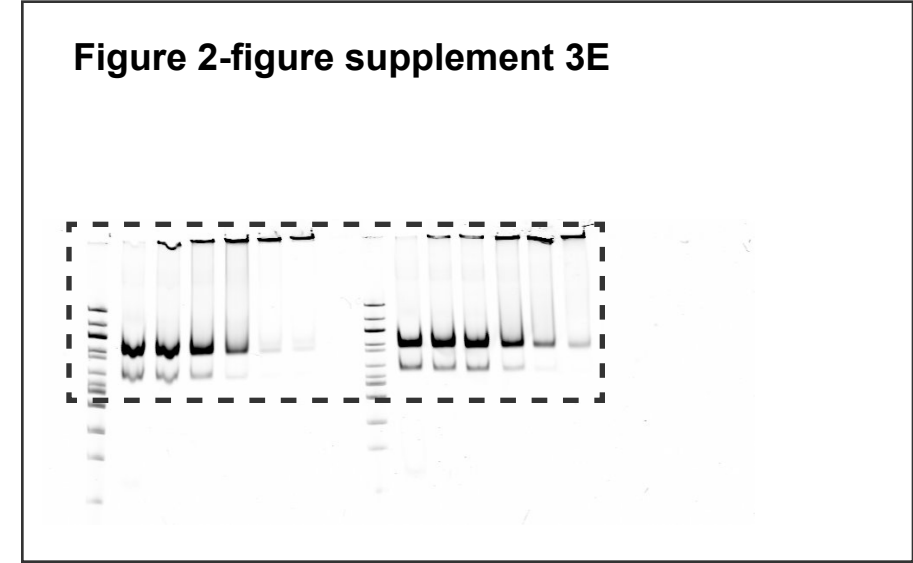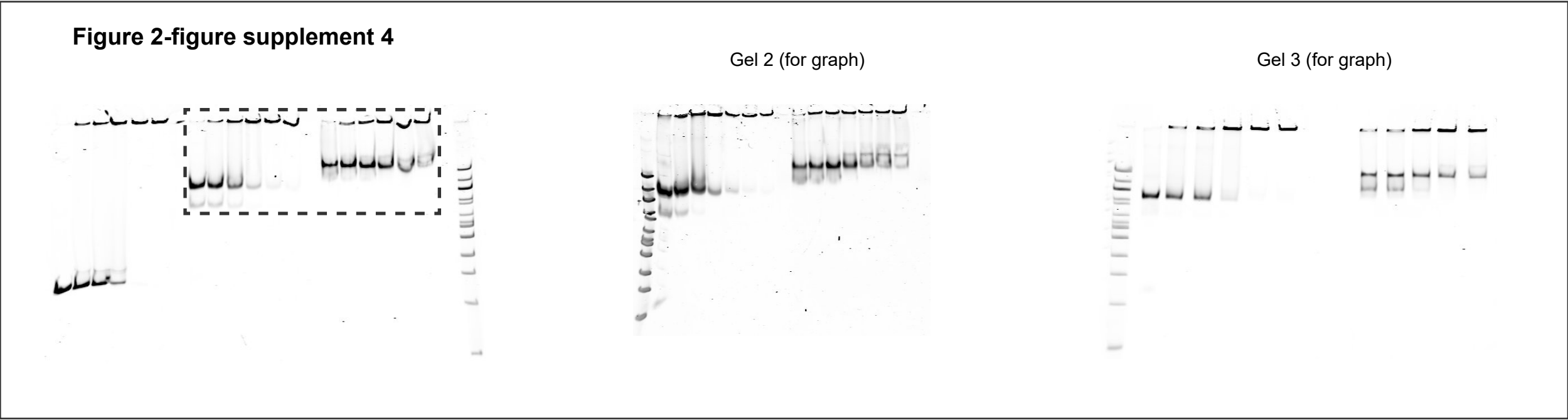

Supplement: Figure 2—source data 1. [file elife-68918-fig2-data1.zip › Fig 2/Fig2_gels.pdf]

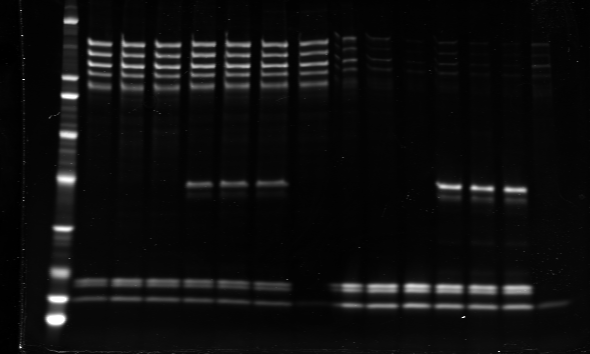

Supplement: Figure 2—source data 1. [file elife-68918-fig2-data1.zip › Fig 2/Figure 2B_1.tif]

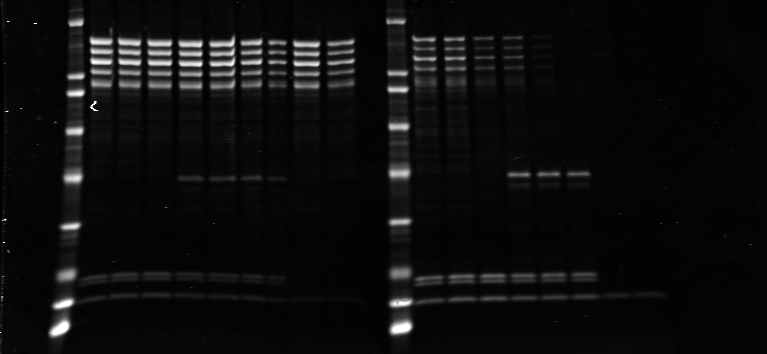

Supplement: Figure 2—source data 1. [file elife-68918-fig2-data1.zip › Fig 2/Figure 2B_2.tif]

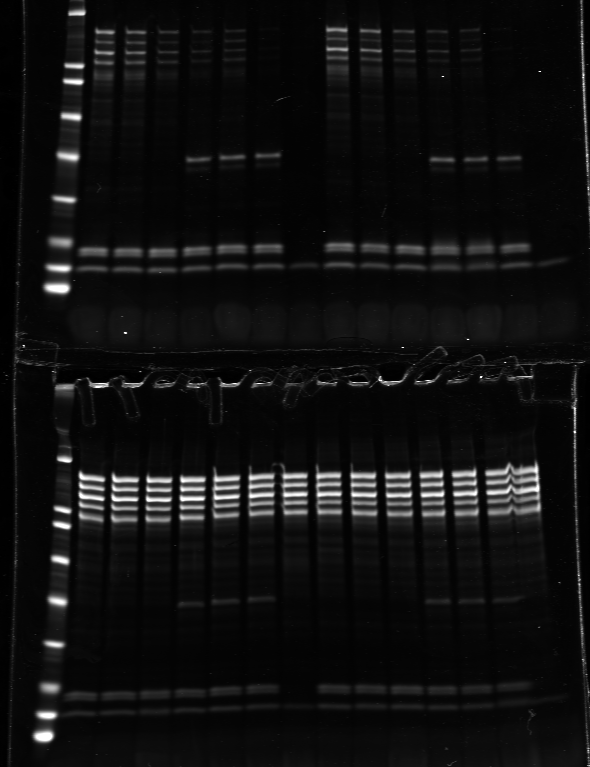

Supplement: Figure 2—source data 1. [file elife-68918-fig2-data1.zip › Fig 2/Figure 2B_3.tif]

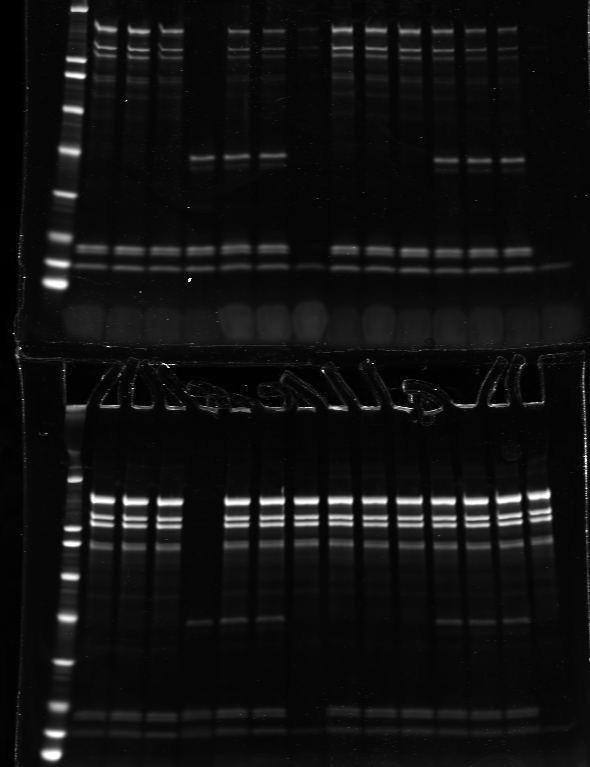

Supplement: Figure 2—source data 1. [file elife-68918-fig2-data1.zip › Fig 2/Figure 2C_1.tif]

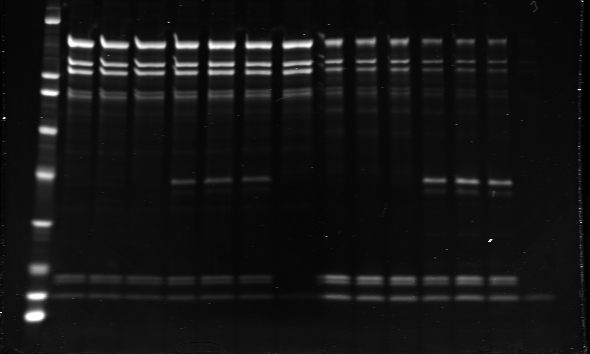

Supplement: Figure 2—source data 1. [file elife-68918-fig2-data1.zip › Fig 2/Figure 2C_2.tif]

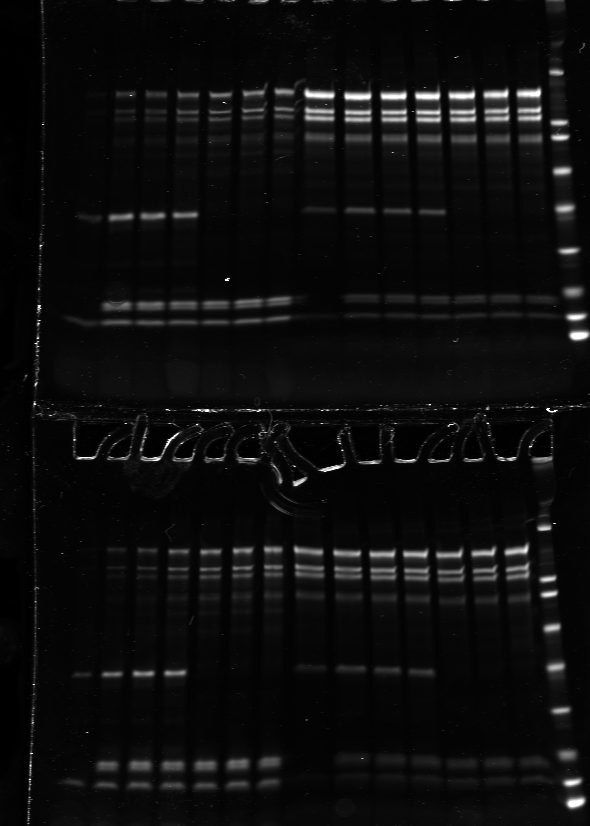

Supplement: Figure 2—source data 1. [file elife-68918-fig2-data1.zip › Fig 2/Figure 2C_3.tif]

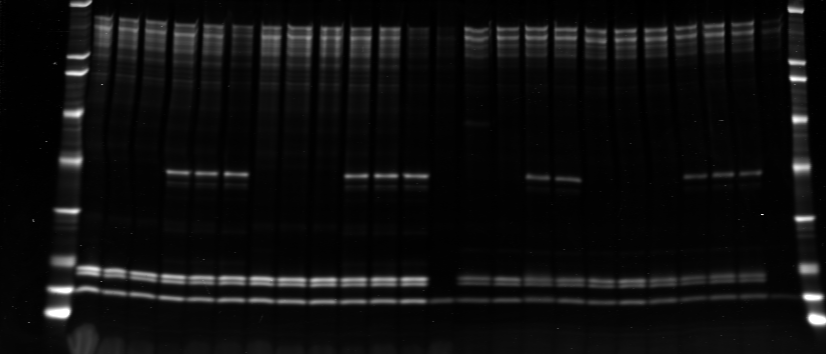

Supplement: Figure 2—source data 1. [file elife-68918-fig2-data1.zip › Fig 2/Figure 2D_1.tif]

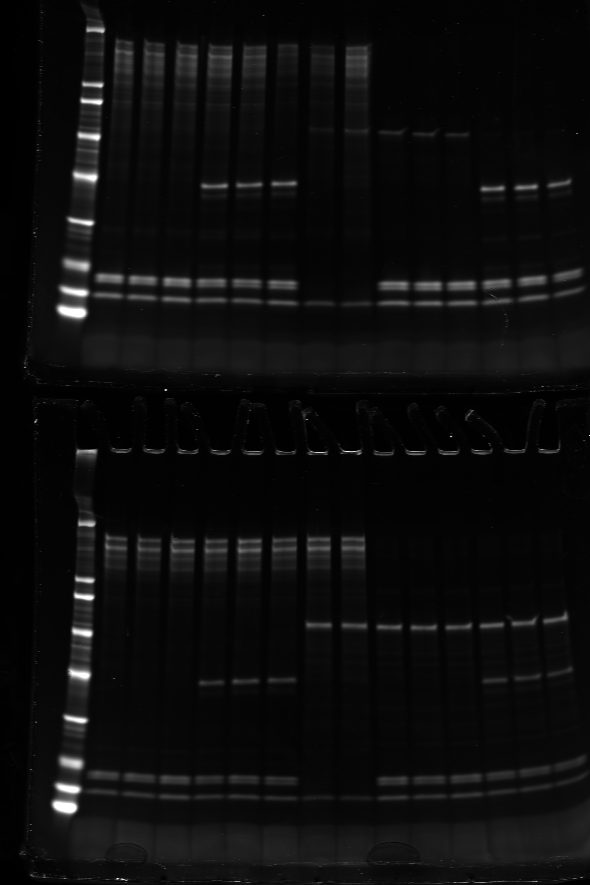

Supplement: Figure 2—source data 1. [file elife-68918-fig2-data1.zip › Fig 2/Figure 2D_2.tif]

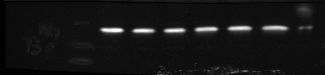

Supplement: Figure 2—source data 1. [file elife-68918-fig2-data1.zip › Fig 2/Figure 2-figure supplement 2A_H3.tif]

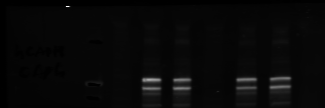

Supplement: Figure 2—source data 1. [file elife-68918-fig2-data1.zip › Fig 2/Figure 2-figure supplement 2A_hCAPG.tif]

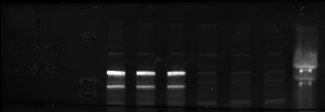

Supplement: Figure 2—source data 1. [file elife-68918-fig2-data1.zip › Fig 2/Figure 2-figure supplement 2A_xCAPG.tif]

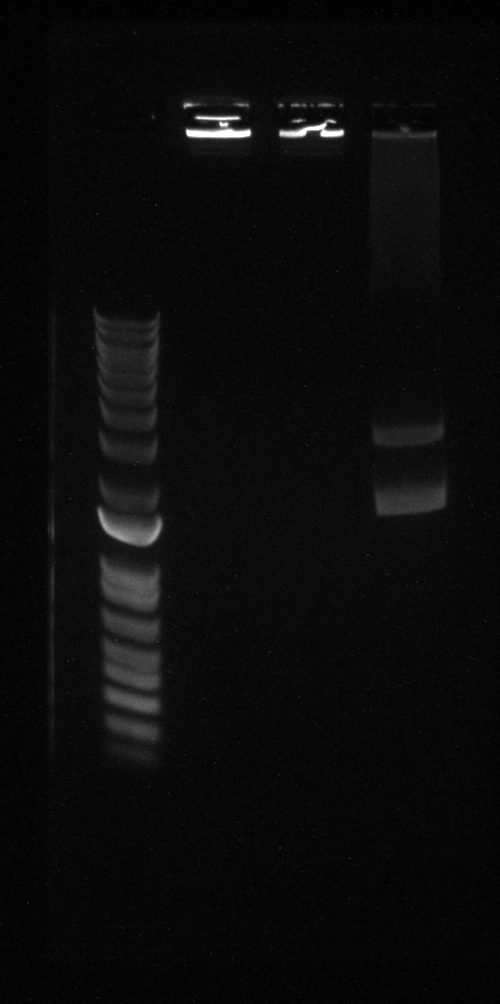

Supplement: Figure 2—source data 1. [file elife-68918-fig2-data1.zip › Fig 2/Figure 2-figure supplement 2B.tif]

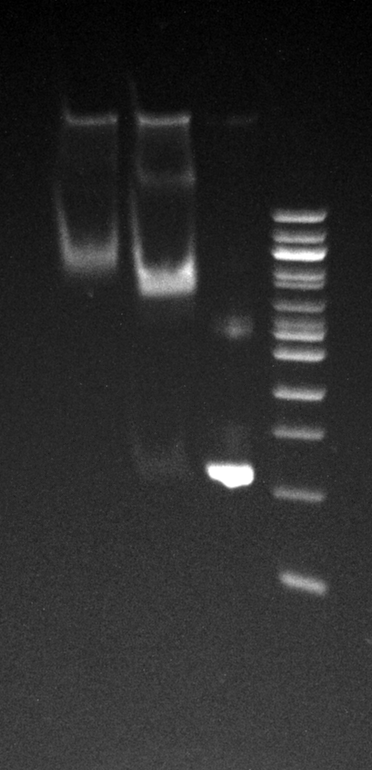

Supplement: Figure 2—source data 1. [file elife-68918-fig2-data1.zip › Fig 2/Figure 2-figure supplement 3A.tif]

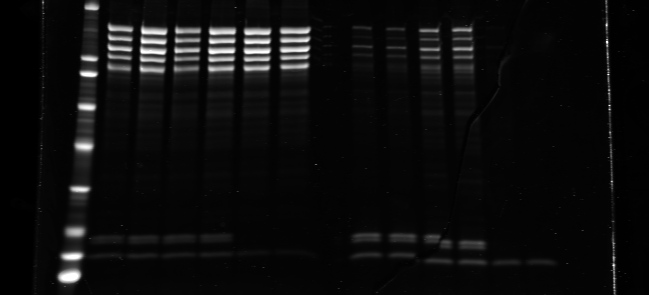

Supplement: Figure 2—source data 1. [file elife-68918-fig2-data1.zip › Fig 2/Figure 2-figure supplement 3B.tif]

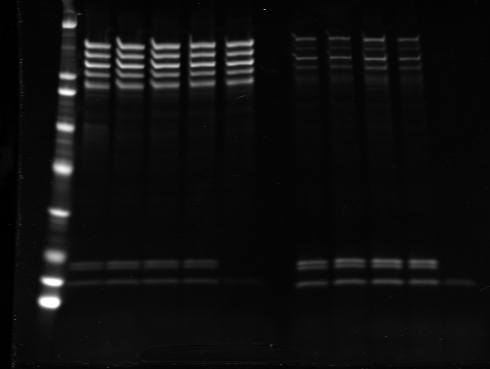

Supplement: Figure 2—source data 1. [file elife-68918-fig2-data1.zip › Fig 2/Figure 2-figure supplement 3C.tif]

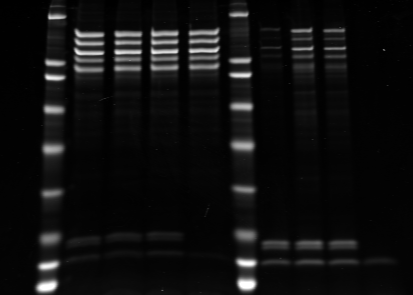

Supplement: Figure 2—source data 1. [file elife-68918-fig2-data1.zip › Fig 2/Figure 2-figure supplement 3D.tif]

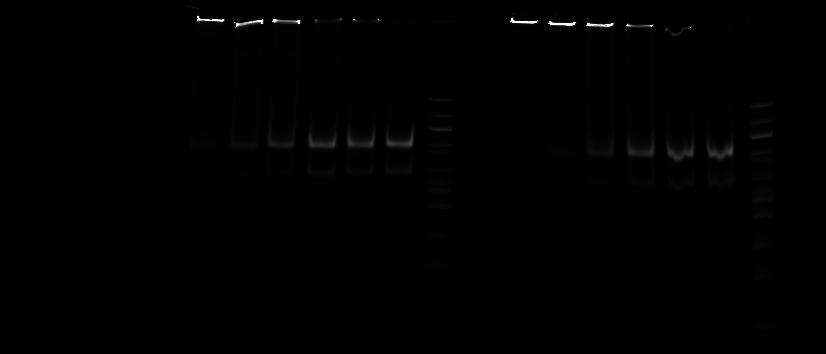

Supplement: Figure 2—source data 1. [file elife-68918-fig2-data1.zip › Fig 2/Figure 2-figure supplement 3E.tif]

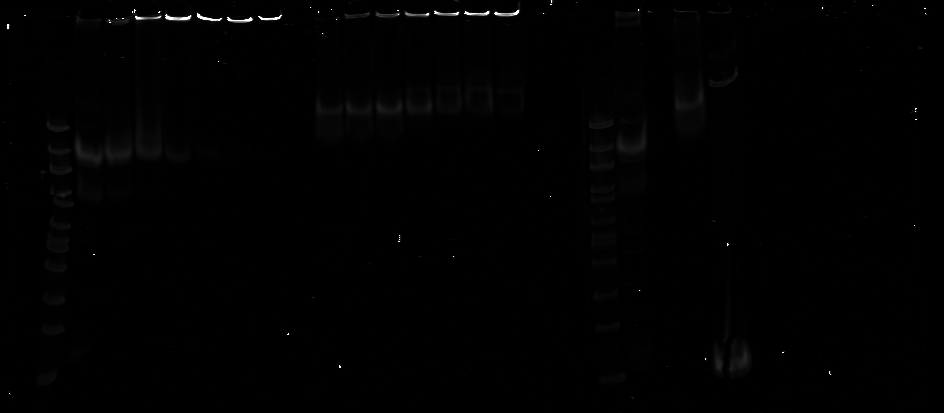

Supplement: Figure 2—source data 1. [file elife-68918-fig2-data1.zip › Fig 2/Figure 2-figure supplement 4_1.tif]

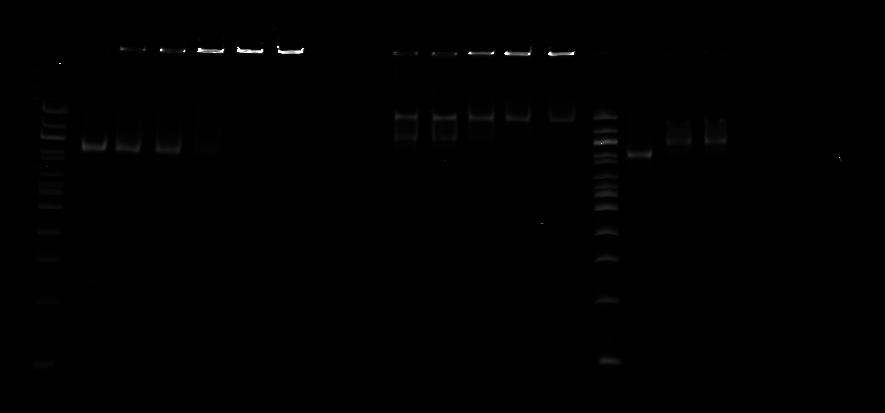

Supplement: Figure 2—source data 1. [file elife-68918-fig2-data1.zip › Fig 2/Figure 2-figure supplement 4_3.tif]

**Figure 3B**

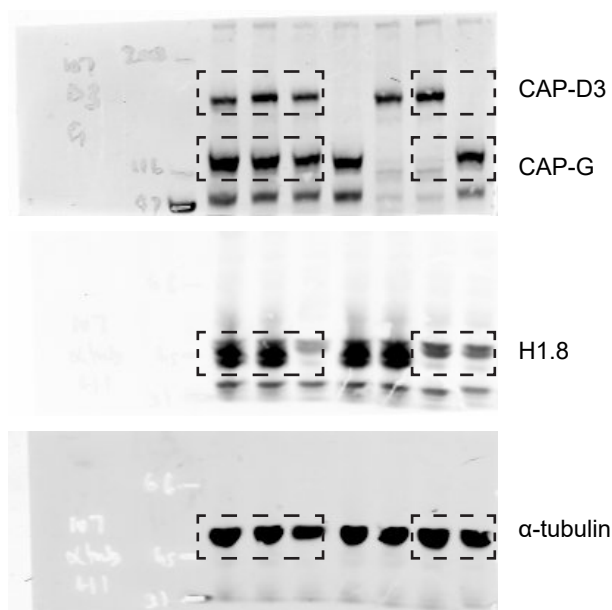

**Figure 3-figure supplement 1B**

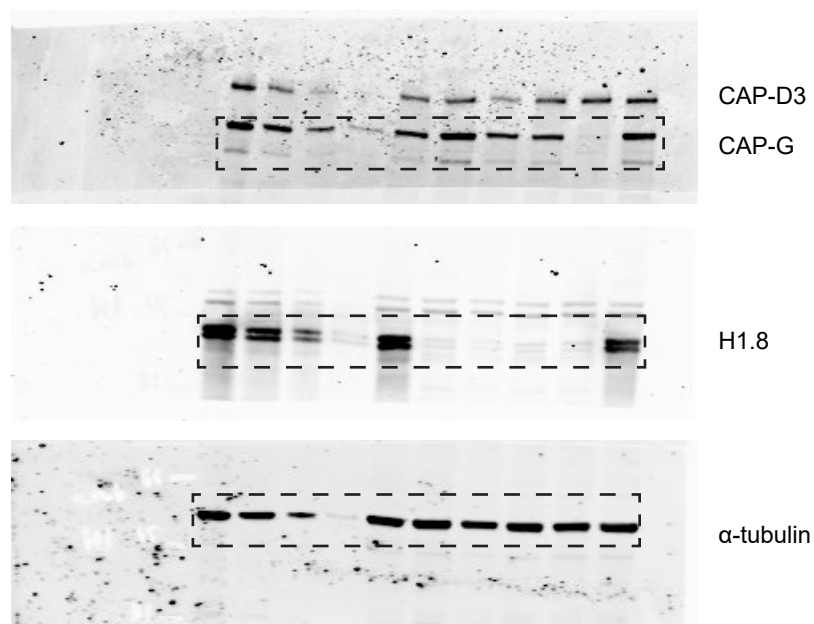

Supplement: Figure 3—source data 1. [file elife-68918-fig3-data1.zip › Fig 3/Figure 3_blots.pdf]

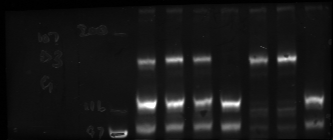

Supplement: Figure 3—source data 1. [file elife-68918-fig3-data1.zip › Fig 3/Figure 3B_CAPG_CAPD3.tif]

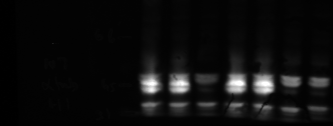

Supplement: Figure 3—source data 1. [file elife-68918-fig3-data1.zip › Fig 3/Figure 3B_H18.tif]

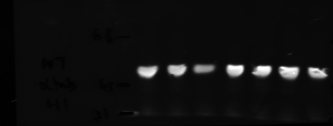

Supplement: Figure 3—source data 1. [file elife-68918-fig3-data1.zip › Fig 3/Figure 3B_tubulin.tif]

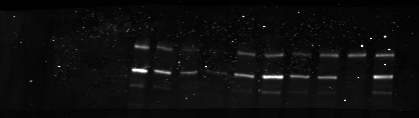

Supplement: Figure 3—source data 1. [file elife-68918-fig3-data1.zip › Fig 3/Figure 3-figure supplement 1B_CAPG_CAPD3.tif]

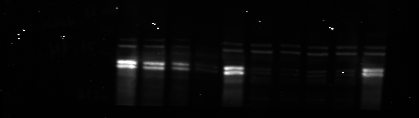

Supplement: Figure 3—source data 1. [file elife-68918-fig3-data1.zip › Fig 3/Figure 3-figure supplement 1B_H18.tif]

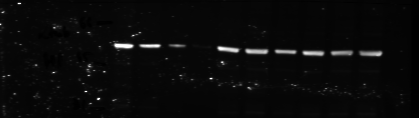

Supplement: Figure 3—source data 1. [file elife-68918-fig3-data1.zip › Fig 3/Figure 3-figure supplement 1B_tubulin.tif]

**Figure 4-figure supplement 2A**

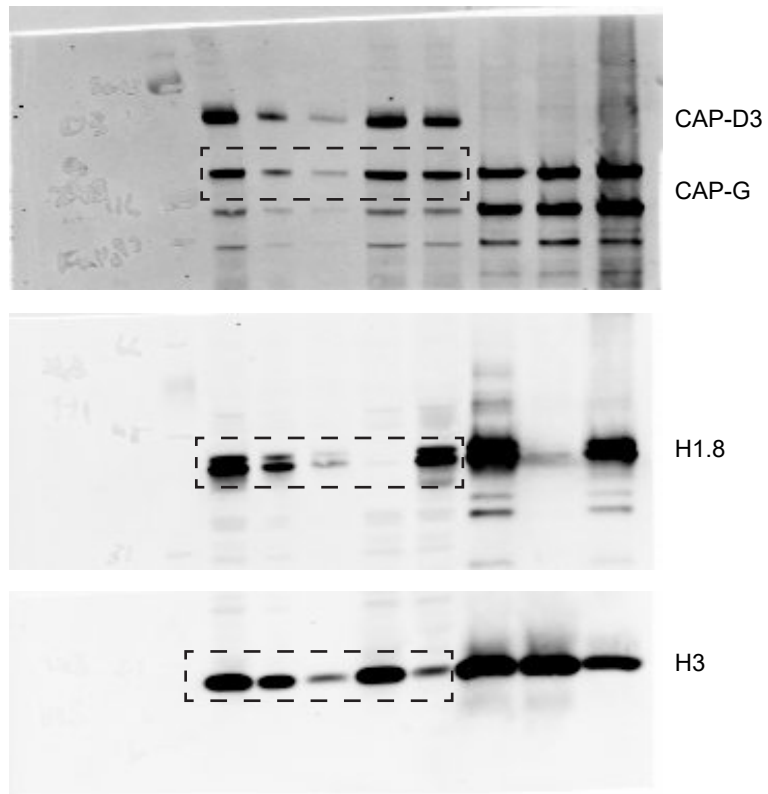

Supplement: Figure 4—source data 1. [file elife-68918-fig4-data1.zip › Fig 4/Figure 4_blots.pdf]

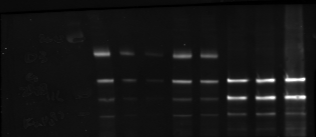

Supplement: Figure 4—source data 1. [file elife-68918-fig4-data1.zip › Fig 4/Figure 4-figure supplement 2A_CAPG_CAPD3.tif]

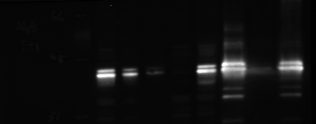

Supplement: Figure 4—source data 1. [file elife-68918-fig4-data1.zip › Fig 4/Figure 4-figure supplement 2A_H18.tif]

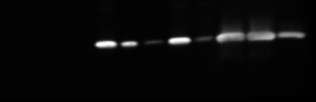

Supplement: Figure 4—source data 1. [file elife-68918-fig4-data1.zip › Fig 4/Figure 4-figure supplement 2A_H3.tif]

**Figure 6A**

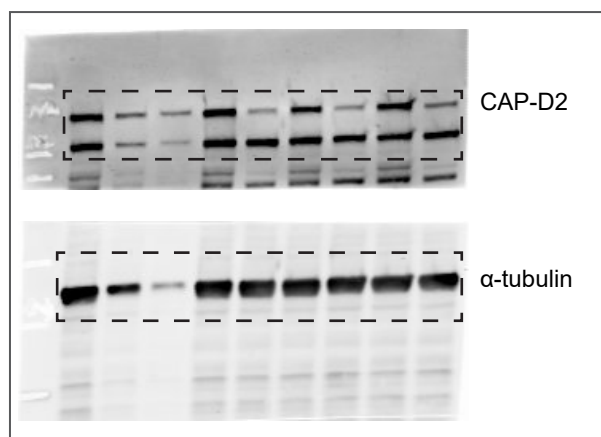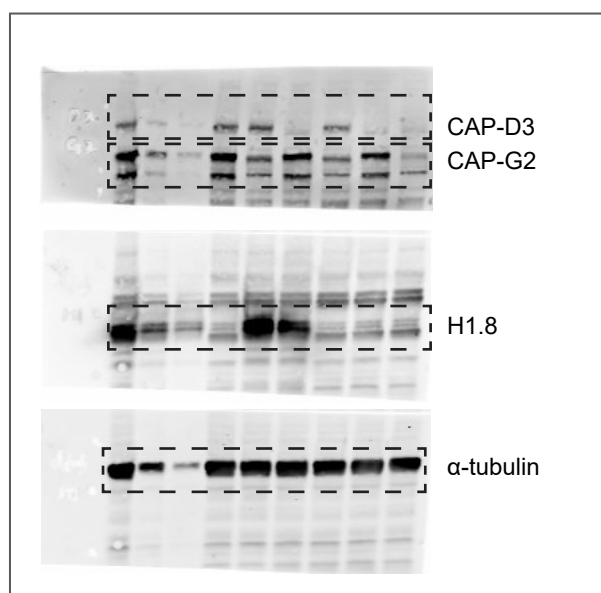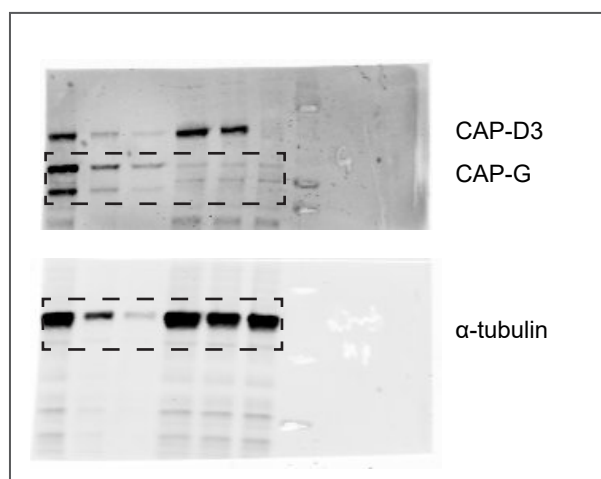

Supplement: Figure 6—source data 1. [file elife-68918-fig6-data1.zip › Fig 6/Figure 6_blots.pdf]

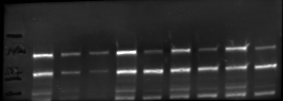

Supplement: Figure 6—source data 1. [file elife-68918-fig6-data1.zip › Fig 6/Figure 6A_CAPD2.tif]

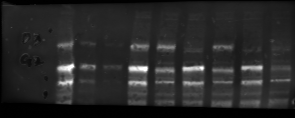

Supplement: Figure 6—source data 1. [file elife-68918-fig6-data1.zip › Fig 6/Figure 6A_CAPD3_CAPG2.tif]

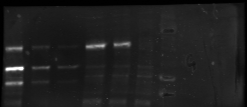

Supplement: Figure 6—source data 1. [file elife-68918-fig6-data1.zip › Fig 6/Figure 6A_CAPG.tif]

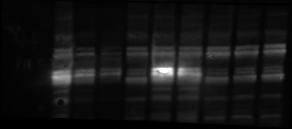

Supplement: Figure 6—source data 1. [file elife-68918-fig6-data1.zip › Fig 6/Figure 6A_H18-CAPD3_CAPG2.tif]

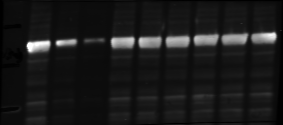

Supplement: Figure 6—source data 1. [file elife-68918-fig6-data1.zip › Fig 6/Figure 6A_tubulin-CAPD2.tif]

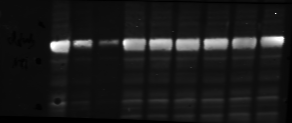

Supplement: Figure 6—source data 1. [file elife-68918-fig6-data1.zip › Fig 6/Figure 6A_tubulin-CAPD3_CAPG2.tif]

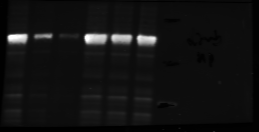

Supplement: Figure 6—source data 1. [file elife-68918-fig6-data1.zip › Fig 6/Figure 6A_tubulin-CAPG.tif]

Figure 7-figure supplement 1A

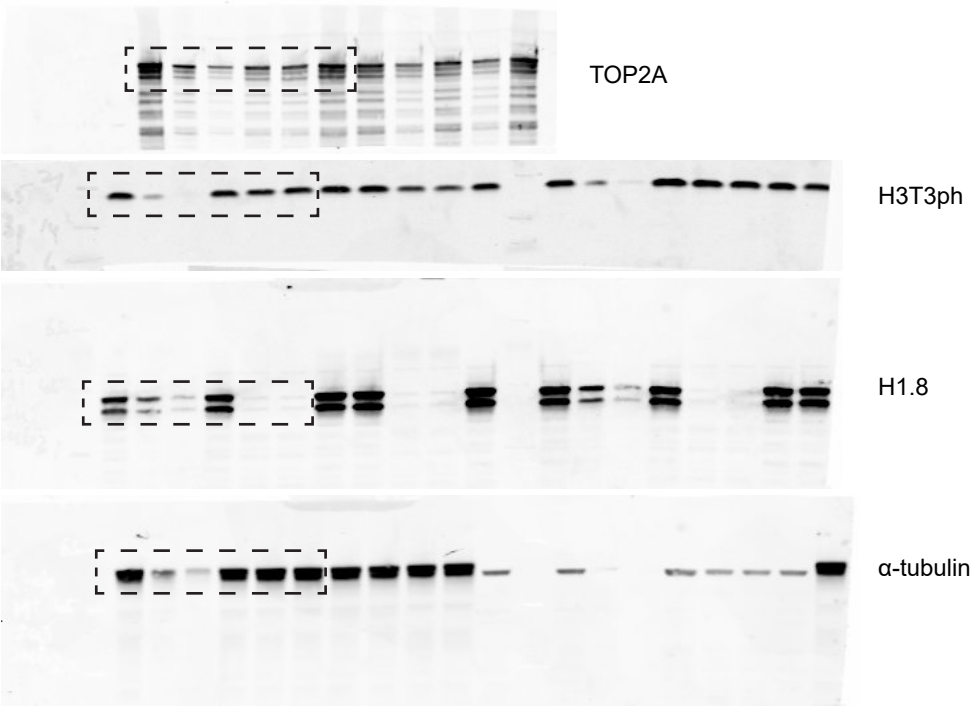

Supplement: Figure 7—source data 1. [file elife-68918-fig7-data1.zip › Fig 7/Figure 7_blots.pdf]

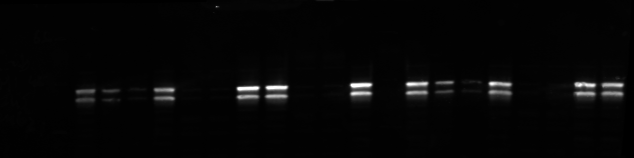

Supplement: Figure 7—source data 1. [file elife-68918-fig7-data1.zip › Fig 7/Figure 7-figure supplement 1A_H18.tif]

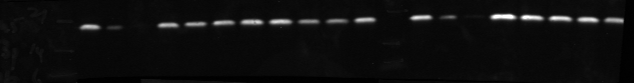

Supplement: Figure 7—source data 1. [file elife-68918-fig7-data1.zip › Fig 7/Figure 7-figure supplement 1A_H3T3ph.tif]

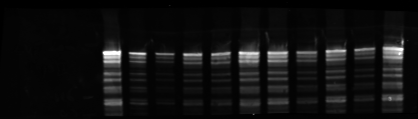

Supplement: Figure 7—source data 1. [file elife-68918-fig7-data1.zip › Fig 7/Figure 7-figure supplement 1A_TOP2A.tif]

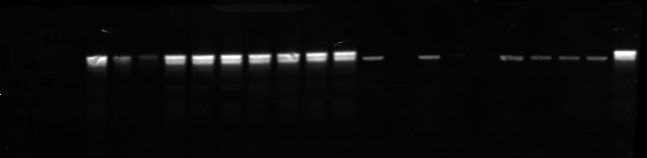

Supplement: Figure 7—source data 1. [file elife-68918-fig7-data1.zip › Fig 7/Figure 7-figure supplement 1A_tubulin.tif]
